# A Judgement Bias Test to Assess Affective State and Potential Therapeutics in a Rat Model of Chemotherapy-Induced Mucositis

**DOI:** 10.1038/s41598-018-26403-7

**Published:** 2018-05-29

**Authors:** Rebecca P. George, Timothy H. Barker, Kerry A. Lymn, Dylan A. Bigatton, Gordon S. Howarth, Alexandra L. Whittaker

**Affiliations:** 1 School of Animal and Veterinary Sciences, University of Adelaide, Adelaide, South Australia Australia; 2 Gastroenterology Department, Women’s and Children’s Hospital, North Adelaide, South Australia Australia

## Abstract

Chemotherapy-induced mucositis is an extremely painful condition that occurs in 40–60% of patients undergoing chemotherapy. As mucositis currently has no effective treatment, and due to the self-limiting nature of the condition, the major treatment aims are to manage symptoms and limit pain with significance placed on improving patient quality of life. Rodent models are frequently used in mucositis research. These investigations typically assess pathological outcomes, yet fail to include a measure of affective state; the key therapeutic goal. Assessment of cognitive biases is a novel approach to determining the affective state of animals. Consequently, this study aimed to validate a cognitive bias test through a judgement bias paradigm to measure affective state in a rat model of chemotherapy-induced intestinal mucositis. Rats with intestinal mucositis demonstrated a negative affective state, which was partially ameliorated by analgesic administration, whilst healthy rats showed an optimistic response. This study concluded that the judgement bias test was able to evaluate the emotional state of rats with chemotherapy-induced mucositis. These findings provide a foundation for future refinement to the experimental design associated with the animal model that will expedite successful transitioning of novel therapeutics to clinical practice, and also improve humane endpoint implementation.

## Introduction

Cancer is a major cause of morbidity and mortality worldwide, with prevalence expected to increase. Consequently, the need to develop diagnostic tools and novel therapeutics has grown. Chemotherapy treatments have vastly improved cancer survival rates, however they are often accompanied by significant side-effects that severely impact patient quality of life. A common, painful and debilitating side-effect is chemotherapy-induced mucositis, which affects the mucous-membrane lining the digestive tract, primarily in the oral cavity and small intestine^1,2^. Mucositis affects approximately 40% to 60% of patients undergoing standard doses of chemotherapy and occurs in up to 100% of patients undergoing high-dose chemotherapy in combination with radiation or hematopoietic stem cell transplant^3–5^. The condition occurs due to the inability of chemotherapy agents to distinguish between neoplastic and normal cells, consequently causing extensive damage^5,6^. The pathobiology of mucositis is described as a five-phase process involving several complex interacting cellular and molecular processes, including clonogenic cell death, activation of reactive oxygen species, ulceration and healing processes^1,7–9^. Typical symptoms include severe abdominal pain due to ulceration, inflammation and deterioration of the mucosal membrane of the gastrointestinal tract. Other symptoms include vomiting, nausea, diarrhoea, dehydration, constipation, bloating, and diminished oral ingestion that can lead to malnutrition and severe weight loss^5^. For neutropenic cancer patients the ulcerative lesions that occur with mucositis pose a greater risk of causing secondary systemic infection that can be potentially life- threatening^8^.

Mucositis is a major dose-limiting factor in cancer treatment, causing possible interruption to treatment regimen by forcing reduction in dose or early termination, rendering it essential to prevent and manage side-effects to the greatest extent possible^8^. As mucositis currently has no effective treatment, and due to the self-limiting nature of the condition, the major treatment aims are to manage symptoms and limit pain with significance placed on improving patient quality of life and the impact on affective state^8,10^. Affective state being a subjective feeling state that encompasses different mood states, such as anxiety, depression, joy or happiness^11^.

The majority of preclinical studies in mucositis have used rodents to investigate pathogenesis of the condition and effectiveness of novel therapeutics. These investigations typically assess pathological outcomes such as gut histological architecture or inflammatory response, yet fail to include a measure of affective state; the key therapeutic goal. Reliable assessment of affective state, integrated with investigation of therapeutic targets, is therefore required to improve translational validity of these models to human patients.

Since animals are incapable of verbally reporting their ‘feelings’, subjective experiences cannot be directly measured. Behavioural and physiological measures have traditionally been used to gauge an animal’s affective state. Although these behavioural and physiological measures provide important information on the arousal of an emotion, they are simplistic and do not provide a complex interpretation of the positive and negative valence of an emotion^12–15^. One such method that has been derived from the human psychology field, and has been used as an indicator of affective state, is assessment of cognitive biases^16^.

Cognitive bias testing is a novel approach to identify emotional states and establish an objective measure of cognitive performance in animals. Despite the promise of cognitive bias assessment methods as indicators of animal affective state these methods are yet to be employed in an animal disease model. It is pivotal for biomedical studies to find reliable assessment tools to evaluate various emotions experienced by animals to enhance animal model refinement, and improve validity of novel therapeutic assessment. Consequently, this study aimed to validate a cognitive bias test through a judgement bias paradigm to measure affective state in a rat model of chemotherapy-induced intestinal mucositis. Further validation of the test was also performed through the addition of an opioid palliative treatment, buprenorphine, which was expected to improve wellbeing.

## Results

### Judgement Bias Test

**Figure 1:**
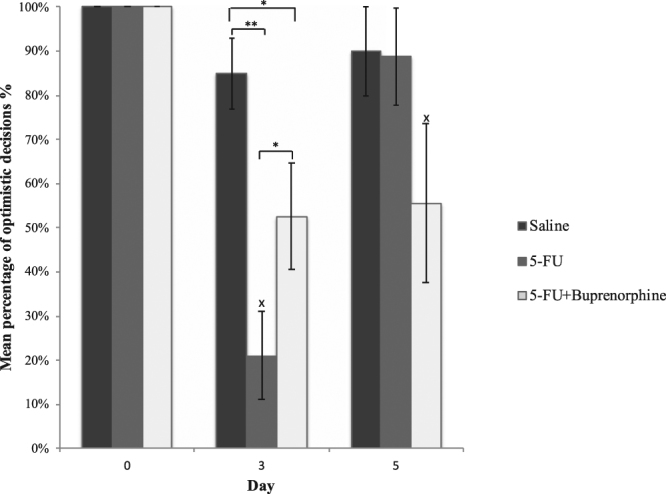
Mean percentage of optimistic decisions on day 0 (baseline prior injection, saline), day 3 (72 hours post injection) and day 5 (120 hours post injection) of rats injected with either saline, 5-FU, and 5-FU + buprenorphine treatments. Data expressed as mean ± estimated SEM. *Indicates significance p < 0.05, **indicates significance p < 0.001. X indicates significance p < 0.05 between time points within the same treatment group.

The results from the judgement bias data indicated that rats administered 5-FU and buprenorphine exhibited significantly fewer optimistic decisions compared to saline alone treated rats (Fig. 1). There were no differences in optimistic decisions between treatment groups prior to administration of saline, 5-FU and buprenorphine (day 0: 100%, p > 0.05). 5-FU-injected rats expressed decreased optimistic decisions compared to animals administered 5-FU + buprenorphine (21%; 53% respectively, p < 0.05) and saline control animals (85%, p < 0.001) 72 hours post injection. There were no differences between treatment groups in the number of optimistic decisions made at the 120 hour time point (saline 90%, 5-FU 89%, 5-FU + buprenorphine 56%, p > 0.05). However, optimistic decisions increased between 72 hour and 120 hour time points for rats administered 5-FU alone (p < 0.001). No differences were detected for rats administered 5-FU in combination with buprenorphine or saline alone between the 72 hour and 120-hour time points (p = 1.000 respectively).

### Daily Bodyweight

**Figure 2:**
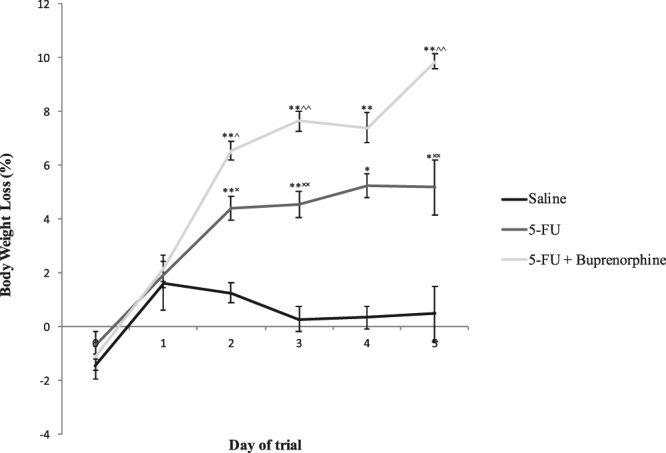
Effects of saline, 5-FU, and 5-FU + buprenorphine on body weight change in SD rats from days 0–5. Bodyweight change calculated from original weight recorded at day 0 prior to saline, 5-FU, buprenorphine injection. Data expressed as mean ± SEM. *p < 0.05, **p < 0.001 compared to saline, ^^^p < 0.05, ^^^^p < 0.001 compared to 5-FU, ^×^p < 0.05, ^××^p < 0.001 compared to 5-FU + buprenorphine.

There were no differences in bodyweight between groups prior to administration of saline, 5-FU and buprenorphine (day 0: p = 0.49; day 1: p = 0.61). Bodyweight change differed substantially between the groups post administration of agents (Fig. 2). Intergroup comparisons demonstrated that rats administered 5-FU alone had an increased bodyweight loss on days 2 to 5 compared to saline control (days 2 and 3: p < 0.001, days 4 and 5: p < 0.05). Furthermore, administration of buprenorphine in conjunction with 5-FU was associated with greater reductions in bodyweight compared to both 5-FU (day 2 p < 0.05, days 3, 4, and 5 p < 0.001) and saline controls (days 2–5 p < 0.001).

### Disease Activity Index

There was no significant difference in disease activity index (DAI) prior to saline, 5-FU or buprenorphine injections (p > 0.05, Table 1). 5-FU administration increased DAI on days 2, 3 and 5 compared to saline controls (p = 0.001, p = 0.003, p = 0.01 respectively). Buprenorphine administration in 5-FU injected rats increased DAI on days 1–5 compared to both saline (day 1 p < 0.05, days 2–5 p < 0.001) and 5-FU controls (days 1 and 5 p < 0.05, days 2–4 p < 0.001).

**Table 1:** Effects of saline, 5-FU, and 5-FU in combination with buprenorphine on disease activity index from days 0–5. Rats were administered saline, 5-FU or 5-FU + buprenorphine on day 1 by intraperitoneal injection.

Day of trial	Saline	5-FU	5-FU + Buprenorphine
0	0	0	0
1	1.0	1.1	1.5*^,^^
2	1.1	1.5*	2.7**^,^^^
3	1.1	1.5*	3.0**^,^^^
4	1.2	1.6	2.1**^,^^^
5	1.1	1.7*	2.6**^,^^

Buprenorphine administration continued at 12 hourly intervals from days 1–5. Data expressed as mean ± SEM. Data are expressed as the mean disease activity index score.

Data expressed on days 0–3 saline n = 20, 5-FU − 5-FU + Buprenorphine n = 19, days 4–5, saline n = 10, 5-FU − 5-FU + Buprenorphine n = 9. *p < 0.05, **p < 0.001 compared to saline, ^^^p < 0.001, ^^^^p < 0.05 compared to 5-FU.

### Histological Severity Score

**Figure 3:**
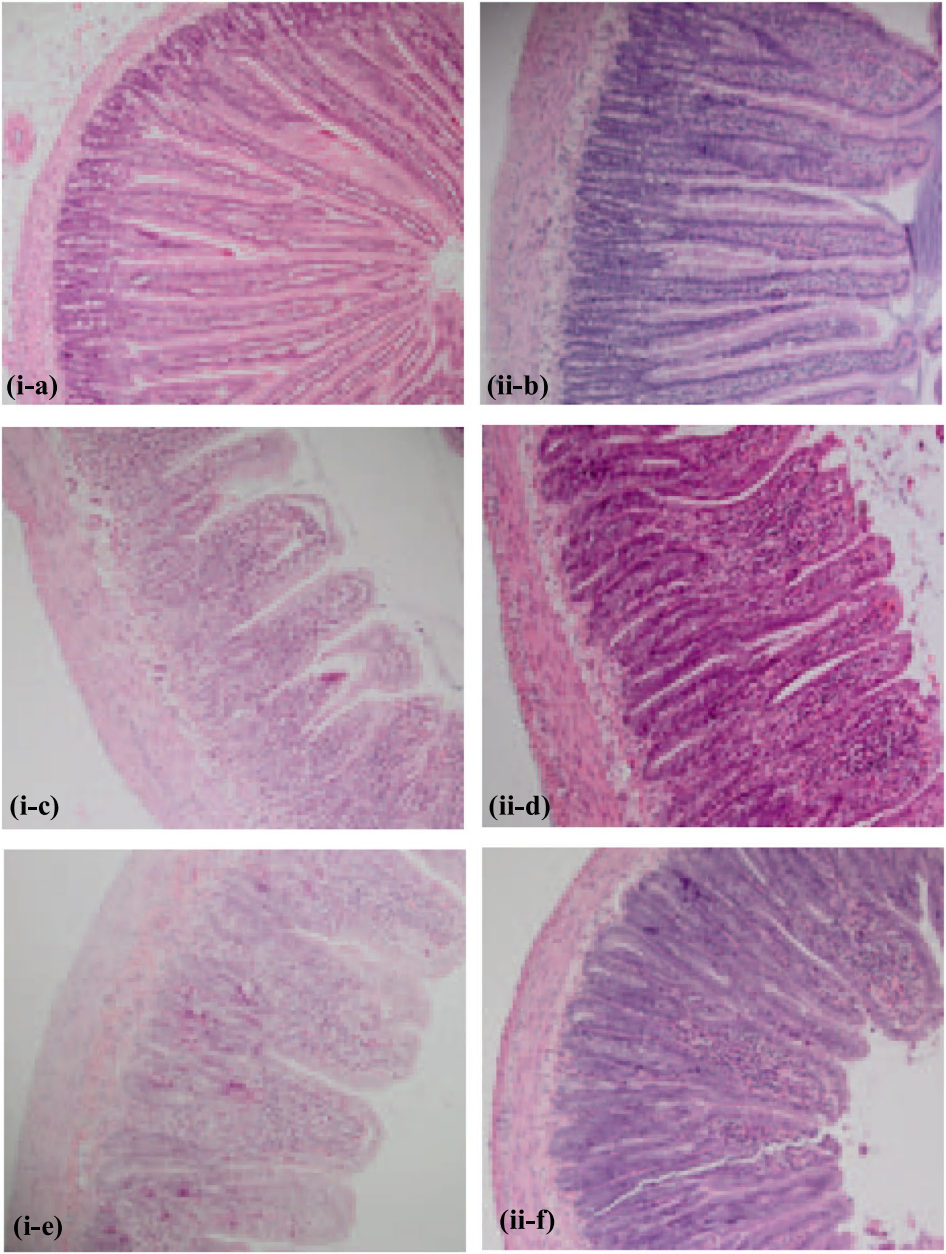
Representative photomicrographs of proximal jejunum at 72 hour (i) and 120 hour (ii) time points stained with haematoxylin and eosin (×40) in rats injected with saline (**a**,**b**), 5-FU (**c**,**d**) and 5-FU + buprenorphine (**e**,**f**).

**Figure 4:**
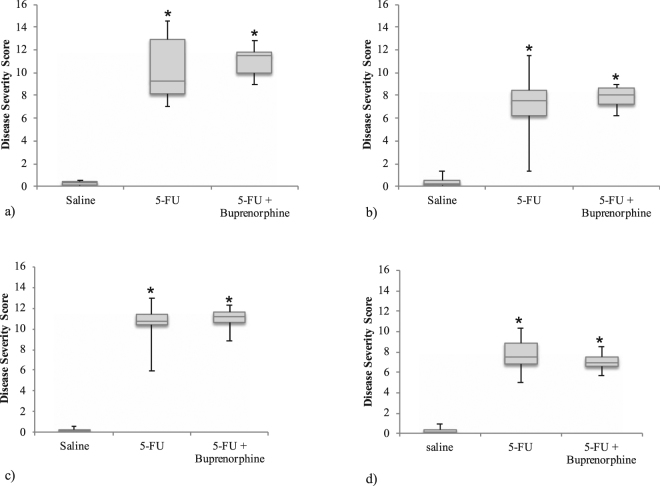
Disease severity score of distal ileum 72 hour (**a**) and 120 hour (**b**) time point and proximal jejunum at 72 hour (**c**) and 120 hour (**d**) time point of rats administered saline, 5-FU and 5-FU + buprenorphine. Data presented as the first and third quartiles, horizontal lines represent the median disease severity score and the whisker ends represent the maximum and minimum score. *Indicates p < 0.001 compared to saline.

Histological gut architecture of the proximal jejunum and distal ileum was consistent with previous literature on mucositis on rats. 5-FU injected rats exhibited an increased histological severity score at 72 hours post injection compared to saline injected rats. Features included decreased goblet cells, crypt and enterocyte disruption, and shortening of villi. A decreased histological severity score was recorded at 120 hours post injection, with the occurrence of villi and crypt elongation (Fig. 3, data not shown for distal ileum). Animals administered 5-FU exhibited an increased disease severity score in the proximal jejunum and distal ileum at both 72 hour and 120 hour, compared to saline injected animals (p < 0.001, Fig. 4). Buprenorphine in conjunction with 5-FU had no effect on histological severity score at 72 hour and 120 hour time points for both ileum and jejunum compared to 5-FU alone treated rats (p < 0.05). Both 5-FU and buprenorphine showed a significant difference from 72 hour and 120 hour time points for jejunum and ileum (jejunum p = 0.005, p < 0.001; ileum p = 0.03, p < 0.001 respectively). This difference was not demonstrated in animals administered saline (jejunum p = 0.90, ileum p = 0.71).

## Discussion

Mucositis is a common and serious side-effect following chemotherapy and radiotherapy. Despite the self-limiting nature of the condition and the negative impact on patient quality of life, cognitive parameters such as affective state are rarely studied in animal models. To our knowledge this is the first study to investigate affective state through judgement biases in a disease model. Importantly, this study represents the first to have demonstrated and validated a reliable judgement bias test to assess affective state exhibited by rats with chemotherapy-induced mucositis. The results from this study indicated that the presence of intestinal mucositis caused a negative affective state, which was partially ameliorated by analgesic administration.

In the present study, judgement bias results followed a correlation with the pathophysiological progression of mucositis identified from previous studies which have evaluated histology and clinical score^6,17^. Healthy animals, prior to 5-FU and saline injections were in a positive affective state, evidenced by optimistic decision-making by all animals when exposed to the ambiguous probe. Administration of 5-FU significantly decreased optimistic decisions compared to healthy controls 72 hours post injection. This finding of negative affective state showed a correlation with the progression of pathological damage associated with mucositis development, where clinical scoring and histological assessment revealed increased histological severity score, decreased bodyweight and increased disease activity index parameters following 5-FU administration. This finding is consistent with previous literature in rats, in which 5-FU caused mucosal damage in the small intestine, villus blunting and fusion, intestinal inflammation characterized by infiltration of immune cells, increased intestinal wall thickness, and increased bodyweight change and clinical disease score at 72 hours post 5-FU injection^6,18^.

Changes in affective state were observed at 120 hours post 5-FU administration. Optimistic decisions increased across time points in animals with mucositis, implying that the healing process of mucositis may have led to an increase in positive affective state. This was evident in the current study since histological severity score decreased with accelerated repair in the damaged intestine 120 hours post 5-FU injection. Histological and disease severity parameters in previous studies have shown evidence of a healing stage 120 hours post 5-FU administration in rats, as gastrointestinal tissue damage subsided, indicated by cell hypertrophy observed via compensatory crypt and villi elongation in the ileum and jejunum, myeloperoxidase activity normalization, and reduction of disease activity index parameters^6,19^.

Interestingly, the analgesic agent, buprenorphine, failed to ameliorate the negative impact on affective state across the 72 hour and 120 hour time points. Differences over time were only present in rats treated with 5-FU alone. Whilst this result appears paradoxical given the commonplace use of opioids to treat severe pain, their use is hampered by side-effects including nausea, respiratory depression, constipation, and urinary retention^20,21^. The associated side-effects of buprenorphine exhibited in rodents are likely playing a part in this judgement bias observation. Side-effects of buprenorphine in rats include; appetite suppression, decreased body weight, and pica behaviour^21,22^. In the current study, buprenorphine had a striking effect on the daily disease activity parameters and bodyweight which is consistent with previous literature^17^. Rats administered 5-FU alone or in combination with buprenorphine showed a significant decrease in bodyweight compared to saline control groups. This decrease in bodyweight was further potentiated throughout the duration of the study in animals administered buprenorphine compared to 5-FU alone. The loss of bodyweight, and side-effects of buprenorphine, most likely had a substantial impact on affective state. These clinical signs are a potential cause of the decreased optimistic behaviour observed across time points when the recovery phase of mucositis was underway.

However, there is an alternative theory for the negative affective state recorded across the 72 hour and 120-hour time points. The repeated administration by injection, and the associated physical restraint used to administer buprenorphine may have had a substantial impact on affective state. A study by Stuart and Robinson^23^ demonstrated that conventional animal restraint used during intraperitoneal substance administration provoked a negative affective state in male Lister Hooded rats. In the current study the route of administration was subcutaneous and the restraint method used was scruffing. Whilst restraint stress was likely present in our study, the subcutaneous restraint method is expected to have had minimal impact compared to restraint methods used during intraperitoneal administration. Nonetheless, repeated restraint stress has previously been applied to cause depressive-like phenotypes and induce negative affective state in rats^23–25^. This is a limitation of the current study, and further research is required to investigate the effects of analgesics, subcutaneous administration and restraint stress on affective state. Through addition of further groups to include an analgesic alone treatment group, saline injection group, and groups utilising alternative methods of analgesic administration such as oral delivery, it would be possible to tease apart the contribution of restraint and injection technique on affective state. Future study design would also benefit from inclusion of additional methods of assessing affective state such as the conditioned place preference test.

There is growing recognition of the limitations associated with animal models, and the challenges to translate outcomes from animal research to medical practice. In clinical studies changes in quality of life and affective state have been associated with cancer treatments^26^. Increase in negative affective state caused by chemotherapy has the potential to exacerbate treatment side-effects^11,27^. Whilst animal models in mucositis research are a valuable tool, they are not without limitations and complications and outcomes do not always translate to the clinic. Key limitations include; dose and scheduling issues (dose rates do not translate to humans), the absence of an emetogenic reflux in rats, impact on drug clearance and toxicities due to sex and strain differences in metabolic enzyme profiles, and difficulties assessing emotional states and other affective behaviours^14,28^. In previous studies the most common indicators used to assess disease progression and wellbeing in mucositis models have been non-specific clinical scoring, bodyweight change and histological analysis. These measures have drawbacks of being retrospective, and the possibility of not indicating true emotional experience^29^. Measuring judgement biases provides a clearer understanding of positive and negative emotional valence. Judgement bias assessment could also be utilised to improve humane endpoint implementation, thus improving animal welfare.

Whilst research into psychiatric disease has seen cognitive bias assessment utilised as an important tool, other biomedical areas of research are yet to incorporate this strategy into disease models. Results from this study demonstrate that the judgement bias test utilised was efficacious to evaluate the emotional state of rats with chemotherapy-induced mucositis. Furthermore, these findings provide a foundation for future biomedical research to incorporate cognitive bias methodologies such as a judgement bias test to determine effectiveness of novel therapeutics, and the mechanisms by which emotion can influence cognitive processes in animal models. Refinement to the experimental design associated with use of these animal models will likely expedite successful transitioning of novel therapeutics to clinical practice.

## Methods

### Animals and Experimental Design

Male Sprague Dawley rats (ArcCrl:CD(SD)IGS, n = 60) were acquired from a specific pathogen-free, barrier-maintained animal facility (Laboratory Animal Services, University of Adelaide, Adelaide, South Australia). Male SD rats were selected due to evidence from previous literature that this strain could be successfully trained using cognitive bias methodologies^12,30,31^. Upon arrival rats were housed in standard open-top cages (415 mm × 260 mm × 145 mm, Tecniplast, Exton, PA, USA) in groups of three, maintained in a room temperature of 21–23 °C with a 12 hour reversed light/dark cycle. All cages were supplied with shredded paper and fibre cycle bedding (Animal Bedding, Fibrecycle Pty Ltd, Queensland, Australia). Food (standard rat chow, Rat and Mouse Cubes, Specialty Feeds, WA, Australia) and RO water was provided at *ad libitum*. All experimental protocols were performed during the dark phase, under red lighting.

Rats were randomly allocated into three experimental groups; saline ip (n = 20); 5-fluorouracil (5-FU) (n = 19) (150 mg/kg 5-FU ip;Mayne Pharma Pty, Ltd, Mulgrave, Vic, Australia); and 5-FU + buprenorphine (n = 19) (150 mg/kg 5-FU ip + 0.05 mg/kg buprenorphine q12hr sc). On day 0 all rats were injected intraperitoneally with 5-FU (150 mg/kg) or saline. Rats in 5-FU + buprenorphine treatment group were injected subcutaneously with 0.05 mg/kg buprenorphine. Buprenorphine was administered at 12-hour intervals for the duration of the study. Buprenorphine was chosen, as it is a commonly used analgesic in rodents. It is favoured due to its simple administration, extended action, partial agonist action at the μ-opioid receptor, and effectiveness in various pain models^22^. Judgement bias response, bodyweight and disease activity index data were collected as described below. Rats were humanely euthanised by CO_2_ asphyxiation at two time points; either 72 hours or 120 hours post 5-FU or saline administration in order to assess gut architecture via histology.

Protocols were approved by the University of Adelaide Animal Ethics Committee and conducted in accordance with the Australian code for the care and use of animals for scientific purposes^32^.

### Judgement Bias Test

**Figure 5:**
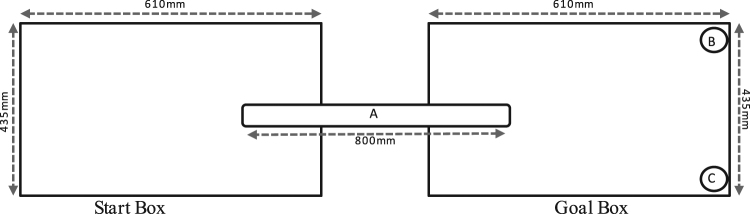
Schematic of the apparatus setup used in the judgement bias test. The apparatus consisted of a start and goal transparent perspex box. The start box was connected to the goal box by a PVC pipe (**a**) that was lined with sandpaper. A blue (**b**) and brown (**c**) reward bowl was placed at the end of the goal box.

Rats were trained using a judgement bias paradigm to distinguish between two tactile stimuli associated with two rewards. The judgement bias paradigm used was based on a previous study by Barker, *et al*.^30^, and consisted of seven phases that included a promotion criterion for each phase (Table 2). The training and testing apparatus consisted of two transparent perspex boxes (610 mm × 435 mm × 215 mm). The start box and goal box were connected with a PVC pipe (100 mm diameter). The inner surface of the PVC pipe was lined with either coarse (P80) or fine (P1200) sandpaper depending on the association. A blue and brown bowl was located at the end of the goal box and contained cinnamon and coriander-scented sand (1% by weight of spice) respectively (Fig. 5). The rewards consisted of a high-positive chocolate reward (Cadbury, London, England) or a low-positive cheerio reward (UncleToby’s, Victoria, Australia). Each rat was randomly allocated a sandpaper association paired with a reward and bowl. During each trial the reward was placed in the bowl and paired with the associated sandpaper. During the training phase, each rat received two chocolate trials and cheerio trials per day. A trial commenced when the rat was placed in the start box and terminated once the rat started to consume the reward or 5 minutes had lapsed. The daily order of these trials and associations was determined independently by randomisation. During the testing phase rats received one ambiguous test, which consisted of the PVC pipe lined with intermediate sandpaper (P180) and no reward being present. Judgement bias was measured by investigating the foraging behaviour (bowl rat first foraged) in response to the ambiguous probe.

**Table 2:** Promotion criteria for each phase of the judgement bias test.

Phase	Description
1	Rats were handled twice daily for a 10-minute duration to become acclimatised to handling. The duration of this phase was five days.
2	Each day rats were tested four times, with test duration of five-minute. Food bowls were located in the testing apparatus, with associated rewards for each individual rat positioned on top of the sand in each bowl. The PVC pipe did not contain any sandpaper. The duration of this phase was five days.
3	Sand paper was positioned in a PVC pipe between the two transparent perspex boxes in the testing apparatus. Each day rats were tested four times, comprised of two cheerio trials and two chocolate trials. The daily order of these trials was determined independently by randomisation. A reward item was positioned in the associated bowl with the associated sandpaper also present. A timer was started once rats were positioned in the start box, and times were recorded for latency to vacate start box, enter goal box, approach a reward bowl, approach the correct reward bowl, and begin to consume reward. Testing ceased once the rat started to consume the reward or when 5 minutes had lapsed. Cleaning of apparatus with seventy percent ethanol solution was performed at the completion of each test. Once rats successfully completed the training on five consecutive days, they were advanced to phase 4.
4	Duplicate protocol to phase 3, except reward items were positioned under the surface of the sand in reward bowls at various depths, and rats were required to unearth reward items. Following successful removal, burial depth of rewards was increased for each succeeding trial, until entirely below the surface. Conditions required for advancement to phase 5 were the same as phase 3.
5	Duplicate protocol to phase 4, except reward items were placed entirely below surface of sand for every trial, and one trial each day selected at random did not contain a reward item. If the first bowl the rat foraged in would normally have contained a reward, the trial was deemed a successful unrewarded trial. Conditions required for advancement to phase 6 were the same as phase 3 and 4.
6	Duplicate protocol to phase 5, except intermediate grade sandpaper was matched with unrewarded trial (P180). The duration of phase 6 was three days.
7	Each day rats were tested once. PVC pipe contained intermediate sandpaper (P180), and food bowls did not contain a reward. The time taken for rats to begin foraging in any bowl was recorded, and record of the first bowl approached and foraged in was taken. The duration of phase 7 was five days.

Once each training phase was successfully achieved, rats entered the test phase and were randomly assigned to an experimental group. Judgement bias was measured 24 hours prior to 5-FU and saline injection to obtain a baseline (day 0), 72 hours post injection (day 3; saline n = 20, 5-FU n = 19, 5-FU + buprenorphine n = 19) and 120 hours post injection (day 5; saline n = 10, 5-FU n = 9, 5-FU + buprenorphine n = 9). Animals that were determined to be in a positive emotional state demonstrated foraging behaviours that would correspond with high-positive reward during the ambiguous trial compared to those in a negative emotional state.

### Disease Activity Index and Bodyweight

Following 5-FU or saline injection DAI and bodyweight were monitored and measured daily to determine the severity of mucositis. DAI was measured on a scale of 0–3 severity per parameter based on rectal bleeding, stool consistency, bodyweight loss, and overall general condition of the animals described by Mashtoub, *et al*.^6^. General condition was determined based on dull or ruffled coat, hunched, pale or sunken eyes, dehydration, squealing when handled and reluctance to move.

### Histological Analysis

Sections (2 cm) of distal ileum and proximal jejunum were collected and fixed in 10% formalin buffer solution. Small intestinal sections were transferred to 70% ethanol 24 h post tissue collection. Tissue samples (4 μm) were processed and embedded in paraffin and stained with haematoxylin and eosin (H&E).

Histological analyses were conducted using a light microscope (Olympus Corporation CX-31, Tokyo, Japan). Histological severity was scored in the jejunal and ileal sections by grading eight histological criteria from zero (normal) to three (maximal damage) in a blinded fashion. This included: enterocyte disruption, reduction in goblet cells numbers, thickening of the submucosa and muscularis externa, villus fusion and stunting, crypt cell disruption, lymphocytic infiltration and crypt disruption^33^.

### Statistical Analyses

Statistical analyses were performed using Megastat Excel Add-In (version 10.3 Release 3.1.6 Mac, McGraw-Hill Higher Education, New York, NY) and SPSS (SPSS Inc., Chicago, IL, USA). Judgement bias data were analysed using a generalised linear mixed model (binary logistic) with the logit link function, where the implicit residual variance was on the underlying scale p^2^/3^34^. The fixed effects were day, treatment, and day x treatment interaction taking into account individual animal to allow for repeated measures. Pairwise comparisons of the estimated marginal means were performed with sequential Bonferroni adjustment. Bodyweight and DAI data were analysed using a repeated measures ANOVA with Tukey’s post hoc test. Histological disease severity score was analysed using a Kruskal-Wallis test with Mann-Whitney U-test. Data were deemed significant at p < 0.05. All data were expressed as means ± standard error of the mean.

### Data Availability

All datasets generated and analysed during this study are included in supplementary information files.
